# Effects of resveratrol on glucose control and insulin sensitivity in subjects with type 2 diabetes: systematic review and meta-analysis

**DOI:** 10.1186/s12986-017-0217-z

**Published:** 2017-09-22

**Authors:** Xiangyun Zhu, Chunhua Wu, Shanhu Qiu, Xuelu Yuan, Ling Li

**Affiliations:** Department of Endocrinology, Affiliated ZhongDa Hospital, School of Medicine, Southeast University, Nanjing, 210009 China

**Keywords:** Resveratrol, Type 2 diabetes, Meta-analysis

## Abstract

Although the regular consumption of resveratrol has been known to improve glucose homeostasis and reverse insulin resistance in type 2 diabetes mellitus (T2DM), the reported results are inconsistent. Thus, we aimed to assess the effects of resveratrol on glycemic control and insulin sensitivity among patients with T2DM. We searched for relevant articles published until June 2017 on PubMed-Medline, Embase, Cochrane Library, and Web of Science. Randomized controlled trials in T2DM patients administered with resveratrol as intervention were included. After study selection, quality assessment and data extraction were performed independently by two authors, and STATA and RevMan software were used for statistical analysis. Nine randomized controlled trials involving 283 participants were included. Meta-analysis showed that resveratrol significantly improved the fasting plasma glucose ( −0.29 mmol/l, 95% CI: −0.51, −0.06, *p* < 0.01) and insulin levels (−0.64 U/mL, 95% CI: −0.95, −0.32, *p* < 0.0001). The drug also reduced homeostasis model assessment of insulin resistance (HOMA-IR) index, systolic blood pressure, and diastolic blood pressure among participants with T2DM. The changes in hemoglobin A1c (HbA1c), low-density lipoprotein cholesterol, and high-density lipoprotein cholesterol were negligible. Subgroup analysis comparing the resveratrol supplementation doses of < 100 mg/d versus ≥ 100 mg/d revealed a significant difference in fasting plasma glucose. In particular, the latter dose presented more favorable results. This meta-analysis provides evidence that supplementation of resveratrol may benefit management of T2DM.

**Electronic supplementary material:**

The online version of this article (10.1186/s12986-017-0217-z) contains supplementary material, which is available to authorized users.

## Background

Type 2 diabetes mellitus (T2DM) is a long-term, multifactorial, metabolic disease with severe complications. Projections indicate that approximately 600 million people will suffer from the disease by 2030 [1, 2]. Increasing morbidity and mortality rates among patients with T2DM are mainly attributed to the high incidence and severity of diabetic complications. These complications pose a major threat to general public health worldwide and lead to high economic costs [3, 4]. Hence, identifying an optimal therapy for T2DM is crucial. Although chemical agents for glycemic control have been adopted in T2DM therapy, these substances are limited by their contraindications and side effects, especially hypoglycemia and weight gain [5], which require an effective treatment method for T2DM.

As a potent antioxidant, resveratrol is a plant-derived polyphenolic compound that possesses anti-inflammatory, anti-platelet aggregation, anti-carcinogenic, cartilage-protective, and anti-aging properties. This compound also improves the endothelial function [6]. Some studies have revealed that resveratrol administration improves insulin sensitivity in diabetic rats and patients with T2DM [7–9]. In vitro and in vivo studies have described resveratrol as a potent activator of histone deacetylase Sirtuin1 (Sirt1) [10, 11]. Sirt1 activation can increase insulin sensitivity and protect against metabolic damage resulting from a high-fat diet. AMP-activated protein kinase (AMPK) activation has been used to mediate some effects of resveratrol in regulating insulin sensitivity and insulin secretion in pancreatic β-cells and increasing glucose uptake [12, 13]. These results indicate that resveratrol is an inexpensive dietary supplement that could benefit T2DM treatment.

Resveratrol was added to the usual therapy in trials, which revealed improvements in glycemic control, insulin sensitivity, and other metabolic parameters of patients with T2DM [14, 15]. However, inconsistent results were on the therapeutic efficacy of resveratrol treatment from several human clinical trials [16, 17]. According to a recent meta-analysis, resveratrol supplementation is more effective than placebo in terms of hemoglobin A1c (HbA1c) and creatinine levels, but this finding is not true for fasting plasma glucose and insulin resistance in patients with T2DM [18]. Furthermore, the evidence supporting the beneficial effects of resveratrol in T2DM treatment is contradicting. In the present work, we therefore performed a meta-analysis of randomized controlled trials (RCTs) to determine whether or not consuming resveratrol could modulate blood glucose homeostasis and improve insulin sensitivity as compared with placebo/control in patients with T2DM.

## Methods

This study follows the Preferred Reporting Items for Systematic Reviews and Meta-Analyses (PRISMA) statement [19]. The PRISMA statement is designed to improve the quality of meta-analyses.

### Literature search

The databases of PubMed-Medline, Embase, Cochrane Library, and Web of Science were searched for RCTs that were published until June 2017 and evaluated the effects of resveratrol treatment versus placebo/control on T2DM. The following search strategy combined free keywords with MeSh terms: [resveratrol or polygonum or polyphenolic compound or red wine or red grapes or knotweed or SRT50] AND [diabetes or diabetic]. Only English language articles were included. A historical search was also performed using the reference lists of relevant articles. The detailed search strategy is presented in Additional file 1: Table S1.

### Study selection

Eligible studies were determined by two reviewers (XYZ, CHW), and disagreement was resolved by discussion and consultation with a third reviewer (SHQ). Inclusion criteria were as follows: (1) published research articles with completed RCTs reported by original articles, (2) participants suffering from T2DM, (3) studies comparing the effects of resveratrol at any dosage with those of placebo/control, and (4) articles on fasting plasma glucose or HbA1c or homeostasis model assessment of insulin resistance (HOMA-IR). Exclusion criteria were as follows: (1) duplicated publications, and only the first publication reporting related outcomes was included; (2) trials involving animals or healthy human subjects; (3) non-randomized trials; (4) case reports or series studies; and (5) articles that provided inadequate information of interest or primary data.

### Data abstraction and quality assessment

The following details of each included trial were extracted to identify the effects of resveratrol on glycemic control: first author’s name, publication year, study design, patient quantity, resveratrol dose, study duration, and outcome measures. The baseline and end point information on glucose parameters, including fasting plasma glucose, fasting insulin, HbA1c, and HOMA-IR, were also extracted. We recorded other indicators (e.g., baseline and end point and changes in systolic blood pressure, diastolic blood pressure, low-density lipoprotein cholesterol [LDL-c], and high-density lipoprotein cholesterol [HDL-c] levels) to thoroughly understand the relationship between cardiometabolic risk indicator and glycemic control. All values were changed to mmol/L for glucose and to pmol/L for insulin by using the conversion factors 1 mmol/L = 18 mg/dL and 1 pmol/L = 6.965 mIU/L, respectively.

We evaluated the bias risk for each study by using the Cochrane tool (Higgins & Green, 2011), which includes random sequence generation, allocation concealment, blinding (participant, personnel, and outcome assessment), incomplete outcome data, selective outcome reporting, and other biases. The judgment of authors is categorized as “Low risk”, “High risk” or “Unclear risk” of bias.

### Publication bias

According to Egger and colleagues, publication bias assessment is not reliable for less than 10 pooled studies [20]. Therefore, in the present study, we could not assess the existence of publication bias by Egger’s test for funnel plot asymmetry.

### Statistical analysis

The primary outcomes were fasting plasma glucose, HbA1c, and HOMA-IR. The secondary outcomes included insulin concentration, systolic blood pressure, diastolic blood pressure, LDL-c, and HDL-c. *I* ^*2*^ statistic and Cochrane’s Q were used to identify heterogeneity among the studies [21]. In case of heterogeneity (Cochrane’s Q *p* < 0.10  or *I* ^*2*^ > 50%), the data were pooled using a random-effect model. Otherwise, the fixed-effect model was used. We calculated the mean differences between resveratrol and placebo/control groups by using the standardized mean difference (SMD) and 95% confidence interval (CI). We extracted the means and standard deviations (SDs) for the baseline and post-treatment for both groups when available. When the means and SDs were unavailable, we extracted the change scores. Subgroup analysis was conducted for fasting plasma glucose in accordance with the following criteria: resveratrol dose at < 100 versus ≥ 100 mg/d; and treatment duration at < 12 weeks versus ≥ 12 weeks. In case of heterogeneity, we performed sensitivity analyses to test the robustness of the pooled estimates, by using the leave-one-out approach (i.e., removing one study each time and repeating the analysis). All the preceding analyses were performed using Stata 12.0 (Stata Corp, Texas, USA) and RevMan v5.2 software.

## Results

### Search results and study characteristics

**Fig. 1:**
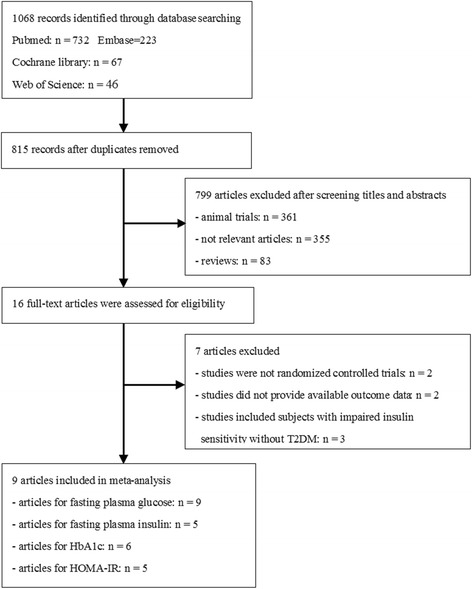
Flow diagram of the study selection procedure showing the number of eligible articles included in the meta-analysis

The initial search yielded 973 potentially relevant articles. Titles and abstracts were screened, and 16 articles were retrieved [7, 14–18, 22–31]. Seven studies were further excluded because (1) two studies were not randomized controlled trials [18, 22], (2) two studies did not provide available outcome data [16, 23], and (3) three studies included subjects with impaired insulin sensitivity but without T2DM [7, 24, 25]. Nine trials involving 283 participants were included in the meta-analysis (Fig. 1) [14, 15, 17, 26–31]. Among these trials, six reported the mean and SD values of HbA1c, five reported the mean and SD values of insulin and HOMA-IR, and all nine reported the mean and SD values of fasting plasma glucose. The sample size range is 10–64 participants. Resveratrol dose ranged from 8 mg/d to 3000 mg/d, and the duration of intervention varied from 4 weeks to 12 months. The detailed characteristics of the studies are presented in Tables 1 and 2.

**Table 1:** Characteristics of the 9 randomized controlled trials included in the analysis

Study	Study design	Population	Duration	Resveratrol group	Control group	Outcomes
Brasnyo et al.	Randomized placebo-controlled double-blinded parallel clinical trial	*N* = 19 patients with T2DM	4 weeks	*N* = 10;10 mg/d	*N* = 9; Placebo	Fasting plasma glucose, fasting insulin, HbA1c, HOMA-IR
Bhatt et al.	Open-label, randomized, controlled trial	*N* = 57 patients with T2DM	3 months	*N* = 28;250 mg/d	*N* = 29; Empty-control	Fasting plasma glucose, HbA1c
Movahed et al.	Randomized placebo-controlled double-blinded parallel clinical trial	*N* = 64 patients with T2DM	45 days	*N* = 33;1 g/d	*N* = 31; Placebo	Fasting plasma glucose, fasting insulin, HbA1c, HOMA-IR
Goh et al.	Randomized double-blind	*N* = 10 patients with T2DM	12 weeks	*N* = 5;3 g/d	*N* = 5; Placebo	Fasting plasma glucose, fasting insulin, HbA1c, HOMA-IR
Tome-Carneiro et al.	Randomized placebo-controlled triple -blinded parallel clinical trial	*N* = 35 patients with T2DM	12 months	*N* = 13; RSV-enriched grape extract (8 mg/d)	*N* = 22; Placebo	Fasting plasma glucose, HbA1c
Bashmakov et al.	Randomized placebo-controlled examiner -blinded parallel clinical trial	*N* = 24 patients with diabetic foot syndrome	60 days	*N* = 14; 50 mg/day	*N* = 10; Placebo	Fasting plasma glucose, fasting insulin
Thazhath et al.	Randomized, double-blind, crossover design	*N* = 14 patients with T2DM	5 weeks	*N* = 14;1 g/d	*N* = 14; Placebo	Fasting plasma glucose, HbA1c
Timmers et al.	Randomized double-blind crossover study	*N* = 17 patients with T2DM	30 days	*N* = 17;150 mg/d	*N* = 17; Placebo	Fasting plasma glucose, fasting insulin, HbA1c, HOMA-IR
Javid et al.	Randomized placebo-controlled double-blinded parallel clinical trial	*N* = 43 patients with T2DM	4 weeks	*N* = 21;480 mg/d	*N* = 22; Placebo	Fasting plasma glucose, fasting insulin, HOMA-IR

*T2DM* type 2 diabetes mellitus, *HOMA-IR* homeostasis model assessment of insulin resistance, *HbA1C* glycated hemoglobin A

**Table 2:** Baseline characteristics of the included studies

Study	Group	Age Mean (SD)	Sex No. of Female/Male	Weight Mean (SD)	BMI Mean (SD)	SBP Mean (SD)	FBG Mean (SD)	Duration– yr. Mean (SD)	Smoker – no. (%)
Brasnyo et al.	Resveratrol Placebo	57·9(7·9) 52.5(11.1)	NA	90.1(16.3) 105.3(16.7)	NA	140(12) 140(17)	7.9(2.21) 8.8(3.2)	NA	0(10) 0(9)
Bhatt et al.	Resveratrol Placebo	56.67(8.91) 57.75(8.71)	16/12 20/9	64.78(9.25) 63.1(9.02)	24.66(3.57) 24.92(3.05)	139.71(16.10) 134.51(14.61)	11.82(3.58) 10.11(2.56)	7.57(4.56) 6.68(4.7)	6/28 6/29
Movahed et al.	Resveratrol Placebo	52.45(6.18) 51.81(6.99)	17/16 16/17	74.26(11.39) 76.60(14.27)	27.05(3.13) 27.83(4.12)	129.03(14.91) 129.31(15.16)	9.76(2.76) 8.40(2.86)	5.81(1.53) 5.39(1.36)	7(21.9) 4.(12.9)
Goh et al.	Resveratrol Placebo	55.8(7.3) 56.3(6.0)	NA	87.0(26.6) 68.3(13.7)	29.4(6.8) 24.4(3.6)	NA	11.8(2.9) 9.5(1.0)	9.4(5.3) 9.6(6.3)	1(10) 2(20)
Tome-Carneiro et al.	Resveratrol Placebo	63(12) 58(10)	NA	84(11) 85(16)	31(5.1) 32(4.5)	130(16) 129(21)	8.39(3) 8.22(2)	NA	2(22) 8(36)
Bashmakov et al.	Resveratrol Placebo	54.0(10.1) 59.8(6.6)	6/8 3/7	NA	28(3.5) 29(2.5)	NA	NA	15(6.9) 15.2(9.5)	3(21) 1(10)
Thazhath et al.	Resveratrol Placebo	NA	NA	81.1(3.7) 81.1(4.3)	NA	NA	8.1(0.3) 8.2(0.3)	NA	NA
Timmers et al.	Resveratrol Placebo	NA	NA	NA	NA	138(11.8) 141(11.7)	7.80(1.62) 7.70(1.70)	NA	NA
Javid et al.	Resveratrol Placebo	49.1(7.4) 50.9(8.9)	16/5 18/4	73.8(10.2) 70.95(11)	29.3(4.9) 28.3(4.8)	NA	8.5(3.1) 9.4(3.0)	NA	NA

*BMI* body mass index, *SBP* systolic blood pressure, *FBG* fasting blood glucose

### Meta-analysis and subgroup analyses of the effects on primary outcomes

#### Fasting plasma glucose

**Fig. 2:**
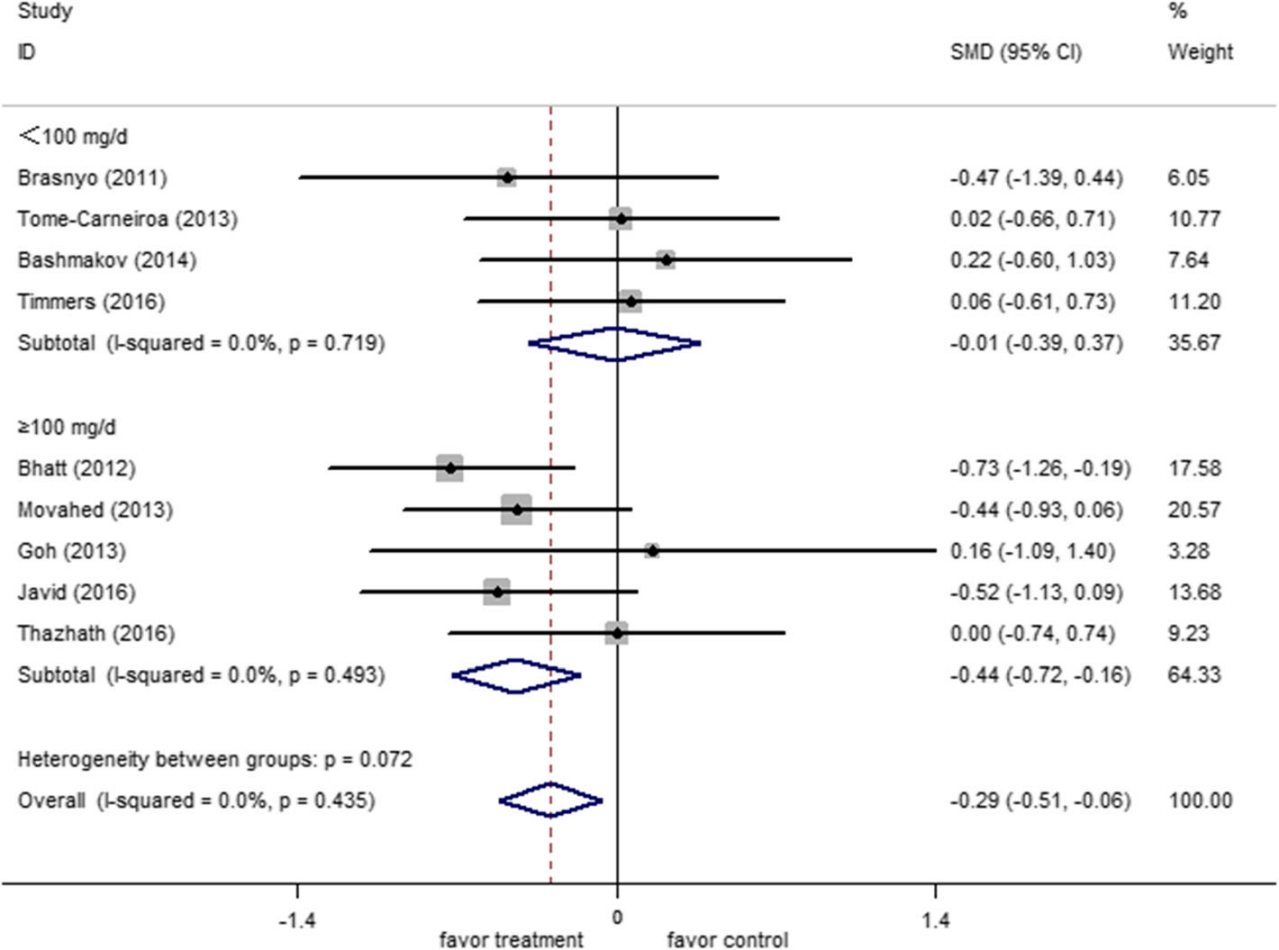
Forest plot of effect of resveratrol on fasting plasma glucose

Nine clinical studies involving 283 participants were included in the analysis to investigate the effects of resveratrol on fasting plasma glucose. A fixed-effect model analysis (*I* ^*2*^ = 0.0%, *p* = 0.44) was performed to pool the data. The overall results of the meta-analysis showed that resveratrol significantly reduced fasting plasma glucose as compared with placebo/control in patients with T2DM (−0.29 mmol/l, 95% CI: −0.51, −0.06, *p* < 0.01) (Fig. 2).

The subgroup analyses showed that fasting plasma glucose was not improve by the low-dose resveratrol (−0.01 mmol/l; 95% CI: −0.39, 0.37; *p* = 0.96), whereas its level significantly declined among the subgroup who received a high dose of resveratrol (−0.44 mmol/l; 95% CI: −0.72, −0.16, *p* < 0.002). The pooled effects of resveratrol on the fasting glucose of the participants were not influenced by the study duration.

#### HbA1c

**Fig. 3:**
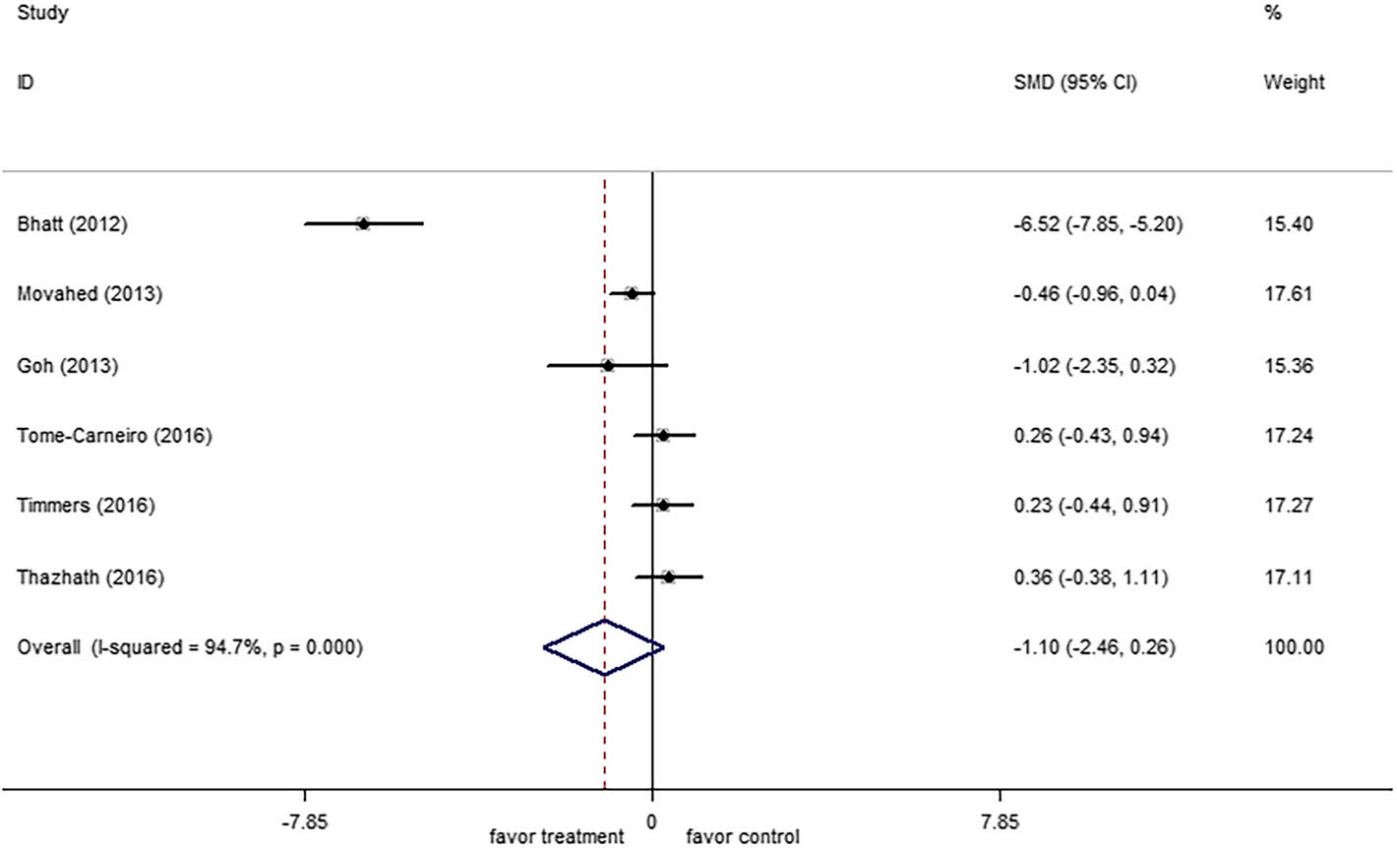
Forest plot of effect of resveratrol on HbA1c

Six studies involving 228 patients reported HbA1c levels before and after intervention. The random-effects model was used because significant heterogeneity was detected (*I* ^*2*^ = 94.7%, *p* = 0.001). The pooled estimates of mean difference suggested no significant difference in the HbA1c level between the resveratrol and control groups (−1.10; 95% CI: −2.46, 0.26; *p* = 0.11) (Fig. 3). When the study by Bhatt et al. [14] was removed, the heterogeneity of study results on HbA1c became insignificant (*I* ^*2*^ = 44.6%, *p* = 0.13). The effect of resveratrol supplementation on HbA1c remained unchanged, thereby suggesting that the study quality does not affect this outcome. The changes in HbA1c level were insignificantly different between the two groups (−0.04; 95% CI: −0.48, 0.39; *p* = 0.13).

#### HOMA-IR

**Fig. 4:**
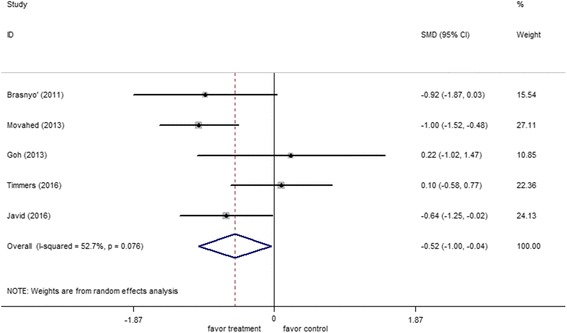
Forest plot of effect of resveratrol on homeostatic model assessment of insulin resistance

Data from five trials involving 153 patients reported the effect of resveratrol therapy on HOMA-IR. Random-effect model analysis (*I* ^*2*^ = 52.7%, *p* = 0.08) was performed to pool the data on HOMA-IR. Meta-analysis of these studies revealed that resveratrol significantly reduced HOMA-IR (−0.52; 95% CI: −1.00, −0.04; *p* < 0.0001) (Fig. 4).

### Meta-analysis of the effects on secondary outcomes

Compared with those in the placebo/control groups, the patients with T2DM who received resveratrol supplementation achieved low insulin levels (−0.64 pmol/L; 95% CI: −0.95, −0.32; *p* < 0.0001), systolic blood pressure (−0.58 mmHg; 95% CI: −0.86, −0.30; *p* < 0.0001), and diastolic blood pressure (−0.43 mmHg; 95% CI: −0.70, −0.15; *p* < 0.003) after treatment. No significantly different effects were observed for LDL-c (−0.57 mmol/l, 95% CI: −2.10, 0.96, *p* = 0.46) and HDL-c (0.30 mmol/l; 95% CI: −0.45, 1.04; *p* = 0.43). An additional movie file shows this finding in detail (Additional file 2: Figure S1).

### Risk of bias assessment

The overall risk of bias in the included trials was moderate. The reporting of allocation concealment was unclear in the majority of trials. Some studies did not provide sufficient information to determine the blinding of the participants, personnel, and outcome assessments. Several studies showed a low bias risk toward selective reporting, and two studies even exhibited a high bias risk in this regard. Three studies exhibited a high bias risk in incomplete outcome data. The quality of bias assessment for the included studies is described in Additional file 3: Figure S2.

### Adverse events

Most studies did not mention adverse reactions during therapy. A major concern in using high dose of resveratrol is related to its toxic effects on the major organs in the body. However, Movahed et al. [28] and Thazhath et al. [17] reported that a maximum dose of 1 g/day of resveratrol is well tolerated and shows no toxic effects in patients with diabetes.

## Discussion

As revealed in this review, resveratrol supplementation for T2DM treatment resulted in significant and clinically important changes in the levels of fasting plasma glucose and insulin, HOMA-IR index, systolic blood pressure, and diastolic blood pressure. However, we failed to show the positive effects on HbA1c, LDL-c, and HDL-c. Subgroup analyses showed a significant effect of high-dose resveratrol supplementation (≥ 100 mg/d) on reducing the fasting plasma glucose levels. By contrast, low-dose resveratrol supplementation (< 100 mg/d) showed no significant effects on fasting plasma glucose levels.

Meta-analysis results are consistent with those of a previous meta-analysis, which reported that resveratrol consumption significantly reduced the fasting glucose and insulin levels, and reversed insulin resistance among participants with diabetes [32]. However, only 3 of the 11 studies included in their review investigated the patients with T2DM. Hausenblas et al. [18] found six eligible studies, which were also included in the present review. In contrast to the findings of the present review, those of the previous review revealed that resveratrol consumption showed that resveratrol has insignificant effects on the fasting glucose and insulin levels but positive effects on HbA1c. However, such study included six trials with <200 participants, which possibly resulted in selection bias. The current systematic review provided additional data that were used to examine the effects of resveratrol treatment on clinically relevant metabolic parameters in patients with T2DM.

Reducing blood glucose levels is a highly important criterion for managing diabetes. Several clinical studies were performed to examine the effectiveness of resveratrol on the hyperglycemia status in patients with T2DM. Most studies consistently reported reduced glucose concentrations. Movahed et al. [28] showed that 1 g/day of resveratrol supplementation for 45 days markedly reduces fasting blood glucose, insulin, and systolic blood pressure. In addition, Goh et al. [27] provided an important evidence to support that resveratrol is a potential glucose-lowering agent in T2DM patients, either through SIRT1 or AMPK activation. However, Faghihzadeh et al. [33, 34] have reported that supplementation of 500 mg of resveratrol does not have any beneficial effect on fasting blood glucose and insulin resistance markers in patients with non-alcoholic fatty liver disease. The most possible explanation is that non-diabetic participants have normal baseline glucose levels and insulin concentrations, and resveratrol consumption may not affect the physiological regulation of plasma glucose and insulin in these subjects. Previous study [32] has shown that resveratrol consumption does not significantly affect the plasma measures of glucose control in non-diabetic participants.

The current meta-analysis results revealed that resveratrol supplementation significantly and positively affected the fasting plasma glucose but not the HbA1c. Fasting plasma glucose and HbA1c exhibited different potentially results because fasting glucose reflects only a time point of glucose metabolism. By contrast, HbA1c, which represents the average levels of plasma glucose over a three-month period, is a marker for long-term glucose control. For example, Thazhath et al. [17] reported that 5 weeks of twice daily 500 mg of resveratrol treatment exerts no effect on HbA1c levels in well-controlled diabetic patients. However, Bhatt et al. [14] showed that 3 months of supplementation with 250 mg of resveratrol modestly reduces HbA1c. In the present work, the HbA1c analysis included only five studies with a small number of subjects and three studies with a short follow-up duration (only 5–6 weeks). This duration may be excessively short to reveal any significant change in the outcome. In addition, a subgroup analysis based on treatment duration could not be performed because of the limited number of studies. Therefore, intervention durations (≥ 12 weeks) might be appropriate for RCTs that assess the effects of resveratrol on glycemic control.

In the present review, the subgroup analyses revealed that high-dose resveratrol (≥ 100 mg/d) supplementation significant reduce the fasting plasma glucose in patients with T2DM. By contrast, the effect of low-dose resveratrol (< 100 mg/d) supplementation was insignificant. These paradoxical results may be attributed to the different resveratrol doses administered in different studies. For instance, the daily administration of resveratrol at doses of 8 [30] and 50 mg [26] did not reveal beneficial effects on metabolic parameter, whereas administration of this drug at higher doses (300, 1000, and 1500 mg) [7, 24, 28] showed beneficial effects on glucose homeostasis. This result suggests a possible direct relationship between the resveratrol dosages and the therapeutic effect. Previous studies indicated that the duration of resveratrol supplementation may influence the outcomes and may differ between preventive and therapeutic clinical studies [6, 32]. Surprisingly, the pooled effects of resveratrol on fasting plasma glucose were not influenced by study duration possibly because of the small sample size.

The HOMA-IR index, a marker of insulin sensitivity, was calculated using fasting insulin and glucose concentrations. Our analysis showed significant reductions in insulin levels and HOMA-IR after resveratrol treatment, thereby suggesting that resveratrol supplementation is beneficial in improving beta cell function and insulin sensitivity and in lowering the insulin levels in patients with T2DM. In accordance with our study, Javid et al. [31] reported a significant decrease in insulin and insulin resistance (HOMA-IR) after resveratrol supplementation for 4 weeks. In *Microcebus murinus*, 33 months of resveratrol treatment improved insulin resistance and glucose tolerance [35]. Experimental and clinical studies suggest that activating inflammatory pathways and oxidative stress may contribute to the pathogenesis of insulin resistance in T2DM. A mechanism that may partly explain the effect of resveratrol on improving insulin sensitivity is the ability of resveratrol to prevent inflammation by improving cellular stress and inhibiting inflammatory gene expression [9, 36]. Resveratrol reduces insulin secretion and thus decreases ATP content and protects the diabetic pancreas from hyperglycemia.

Type 2 diabetics with hypertension and non-high density lipoprotein cholesterol have higher probability to contract atherosclerotic cardiovascular diseases than those with normal variables [37]. The current meta-analysis revealed that the resveratrol supplementation significantly reduced systolic blood pressure and diastolic blood pressure, although such topic has not been systematically studied in hypertensive subjects. The drop in blood pressure has been observed with resveratrol. Timmers et al. [29] reported that 30 days of 150 mg/d resveratrol supplementation reduces systolic blood pressure levels. Furthermore, Bhatt et al. [14] showed the same effect on patients with T2DM. On one hand, resveratrol acutely improves vascular endothelial function and blood flow in humans. On the other hand, small but significant reductions in blood pressure may be attributed to the improvements in glucose homeostasis and insulin resistance. In the current work, we found no significant change in the serum lipid profiles of T2DM patients. Studies in humans and animal models suggest that resveratrol exert beneficial effects on lipids by modulating the genes involved in lipid metabolism [38, 39]. However, an [28] included study reported a positive effect for HDL. All other studies reported insignificant effects for increased HDL levels. Decreasing trends were also observed, but no significant change was noted in the LDL levels after resveratrol supplementation. The effects of resveratrol on lipids were apparent only in obese subjects and not in subjects with low body mass index. Resveratrol could have failed to influence metabolism because most patients were not obese.

Our review features a number of strengths. This review includes all available RCTs addressing the clinical question and is the most up-to-date systematic review of the topic. Our review also considered the dose and duration of resveratrol supplementation in T2DM patients in great detail. Nevertheless, several limitations exist in the present analysis. First, the study used the data provided by the published literature, and the data for each patient were unavailable. Hence, test condition bias might exist. Second, some included studies were of low quality because random allocation schemes were not hidden. Thus, the findings were unreliable. With reduced reliability, the results should be treated with caution in clinical practice. Third, the sample size of the included RCTs was so small that significant metabolic changes associated with resveratrol might not have been detected. Another limitation is that the forms of resveratrol obviously differed. Extracts and powders may provide different bioactive compounds with varying levels of potency and bioavailability. The differences in dose and duration might have also affected the accuracy of the results. Therefore, high-quality studies are required to determine the dose-dependent effects at varying treatment periods.

## Conclusions

This study provides novel insights into the beneficial effects of resveratrol supplementation on T2DM. Specifically, resveratrol supplementation may improve fasting plasma glucose, HOMA-IR, and insulin in diabetic patients. This result proves that the drug may ameliorate metabolic parameters. The dose and/or duration of treatment with resveratrol might also influence the effect of resveratrol on glucose homeostasis. Therefore, studies with durations longer than three months should be designed to confirm the efficacy of resveratrol and determine the appropriate dosage regimen in managing T2DM. Most of the included articles did not explain whether adverse events occurred in the studies. The long-term risks and benefits of resveratrol supplementation are unknown. Understanding the efficacy of resveratrol in diabetic patients requires large-scale, well-designed, and population-based studies in the future.

## Additional files

Additional file 1: Table S1. Search stragegy. (DOCX 16 kb)

Additional file 2: Figure S1(A-E). Funnel plots of meta-analysis of the effect of resveratrol on other parameters. Forest plot of effect of resveratrol on insulin level. Forest plot of effect of resveratrol on systolic blood pressure. Forest plot of effect of resveratrol on diastolic blood pressure. Forest plot of effect of resveratrol on low-density lipoprotein cholesterol. Forest plot of effect of resveratrol on high-density lipoprotein cholesterol. (ZIP 1369 kb)

Additional file 3: Figure S2. Risk of bias of include trials. Random sequence generation: Unclear risk of bias in 4 trails for insufficient information about the sequence generation process. Allocation concealment: Unclear risk of bias for insufficient information in 7 trails. Blinding of participants and personnel: Unclear risk of bias for insufficient information in 3 trails. Blinding of outcome assessment: Unclear risk of bias for insufficient information in 2 trails. Incomplete outcome data: High risk of bias in 3 trails for unbalanced high proportion of dropped participants. Other bias: Low risk of bias in 1 trails. (TIFF 212 kb)

## Abbreviations

BMI: Body mass index
FBG: Fasting blood glucose
HbA1c: Hemoglobin A1c
HDL-c: High density lipoprotein cholesterol
HOMA-IR: homeostasis model assessment of insulin resistance
LDL-c: Low density lipoprotein cholesterol
RCT: Randomized control trial
