# Supplementary material for: Effects of resveratrol on glucose control and insulin sensitivity in subjects with type 2 diabetes: systematic review and meta-analysis
Source: Nutr Metab (Lond). 2017 Sep 22;14:60. doi: 10.1186/s12986-017-0217-z (PMC5610395; doi:10.1186/s12986-017-0217-z)
Supplement: Supplementary file 1 — Search stragegy. (DOCX 16 kb) [file 12986_2017_217_MOESM1_ESM.docx]

# Table S1 Search stragegy

**Pubmed search stragegy**

| set | search | results |
| --- | --- | --- |
| #1  #2  #3  #4  #5 | Search (resveratrol or resveratrols or red wine or red grapes or polygonum or polyphenolic compound or knotweed or SRT501)  Search "Diabetes Mellitus"[Mesh]  Search diabetes[TIAB]  Search (diabetes[TIAB]) OR "Diabetes Mellitus"[Mesh]  Search (((resveratrol or resveratrols or red wine or red grapes or polygonum or polyphenolic compound or knotweed or SRT501))) AND ((diabetes[TIAB]) OR "Diabetes Mellitus"[Mesh]) | [15346](https://www.ncbi.nlm.nih.gov/pubmed/?cmd=HistorySearch&querykey=8)  [359902](https://www.ncbi.nlm.nih.gov/pubmed/?cmd=HistorySearch&querykey=2)  [421799](https://www.ncbi.nlm.nih.gov/pubmed/?cmd=HistorySearch&querykey=1)  [531589](https://www.ncbi.nlm.nih.gov/pubmed/?cmd=HistorySearch&querykey=3)  732 |

**Embase(ovid) search strageg**y

| set | search | results |
| --- | --- | --- |
| #1 | (diabetes or diabetic).mp. [mp=title, abstract, heading word, drug trade name, original title, device manufacturer, drug manufacturer, device trade name, keyword, floating subheading] | 945311 |
| #2 | (resveratrol or resveratrols or red wine or red grapes or polygonum or polyphenolic compound or knotweed or SRT501).mp. [mp=title, abstract, heading word, drug trade name, original title, device manufacturer, drug manufacturer, device trade name, keyword, floating subheading] | 23968 |
| #3  #4 | #1 AND #2  limit #3 to (human and english language and embase status) | 2071  223 |

**Web of Science search strageg**y

| set | search | results |
| --- | --- | --- |
| #1 | Search (diabetes OR diabetic) | 586590 |
| #2 | Search (((((((resveratrol) OR resveratrols) OR red wine) OR red grapes) OR polygonum) OR polyphenolic compound) OR knotweed) OR SRT501 | 70676 |
| #3  #4 | Search #1 AND #2  Search #3 in Clinical Trial | [[2,419](http://apps.webofknowledge.com/summary.do?product=UA&doc=1&qid=6&SID=4B1fRL5pzKtyfecaw7Q&search_mode=CombineSearches&update_back2search_link_param=yes)](http://apps.webofknowledge.com/summary.do?product=WOS&doc=1&qid=9&SID=1A3sEwDeP9jWKwQ2JxO&search_mode=CombineSearches&update_back2search_link_param=yes)  46 |

**Cochrane search strageg**y

| set | search | results |
| --- | --- | --- |
| #1 | diabetes:ti,ab,kw or diabetic (Word variations have been searched) | 48605 |
| #2 | (((((((resveratrol) or resveratrols) or red wine) or red grapes) or polygonum) or polyphenolic compound) or knotweed) or SRT501:ti,ab,kw (Word variations have been searched) | [32,777](http://apps.webofknowledge.com/summary.do?product=WOS&doc=1&qid=41&SID=4BvMX2EgEnDXSncqLhF&search_mode=GeneralSearch&update_back2search_link_param=yes) |
| #3  #4 | #1 AND #2  #4 Trails | 1358  67 |
