# Supplementary figures and images for: Effects of resveratrol on glucose control and insulin sensitivity in subjects with type 2 diabetes: systematic review and meta-analysis
Source: Nutr Metab (Lond). 2017 Sep 22;14:60. doi: 10.1186/s12986-017-0217-z (PMC5610395; doi:10.1186/s12986-017-0217-z)

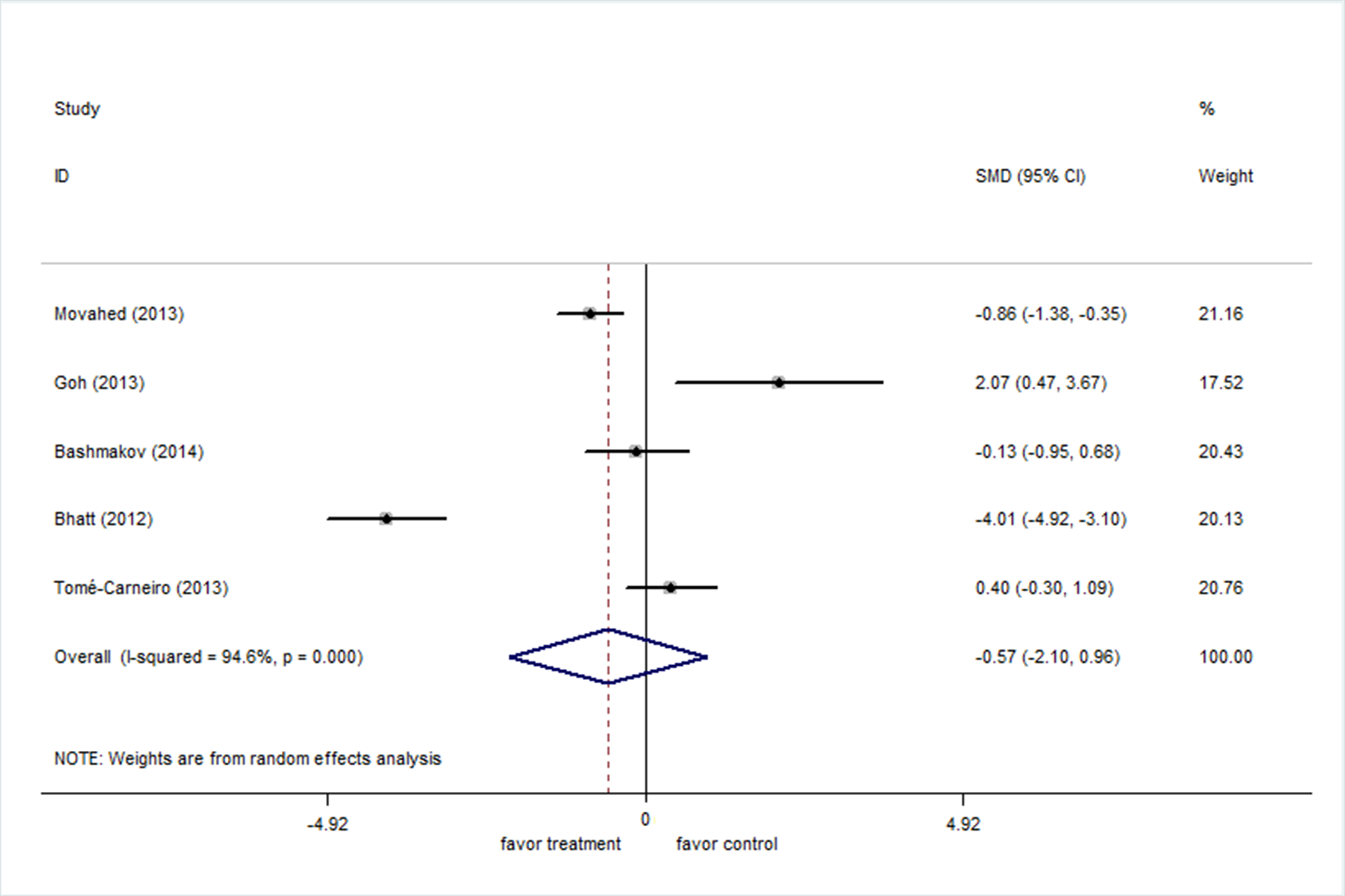

Supplement: Supplementary file 2 — Funnel plots of meta-analysis of the effect of resveratrol on other parameters. Forest plot of effect of resveratrol on insulin level. Forest plot of effect of resveratrol on systolic blood pressure. Forest plot of effect of resveratrol on diastolic blood pressure. Forest plot of effect of resveratrol on low-density lipoprotein cholesterol. Forest plot of effect of resveratrol on high-density lipoprotein cholesterol. (ZIP 1369 kb) [file 12986_2017_217_MOESM2_ESM.zip › Figure_S1D.tif]

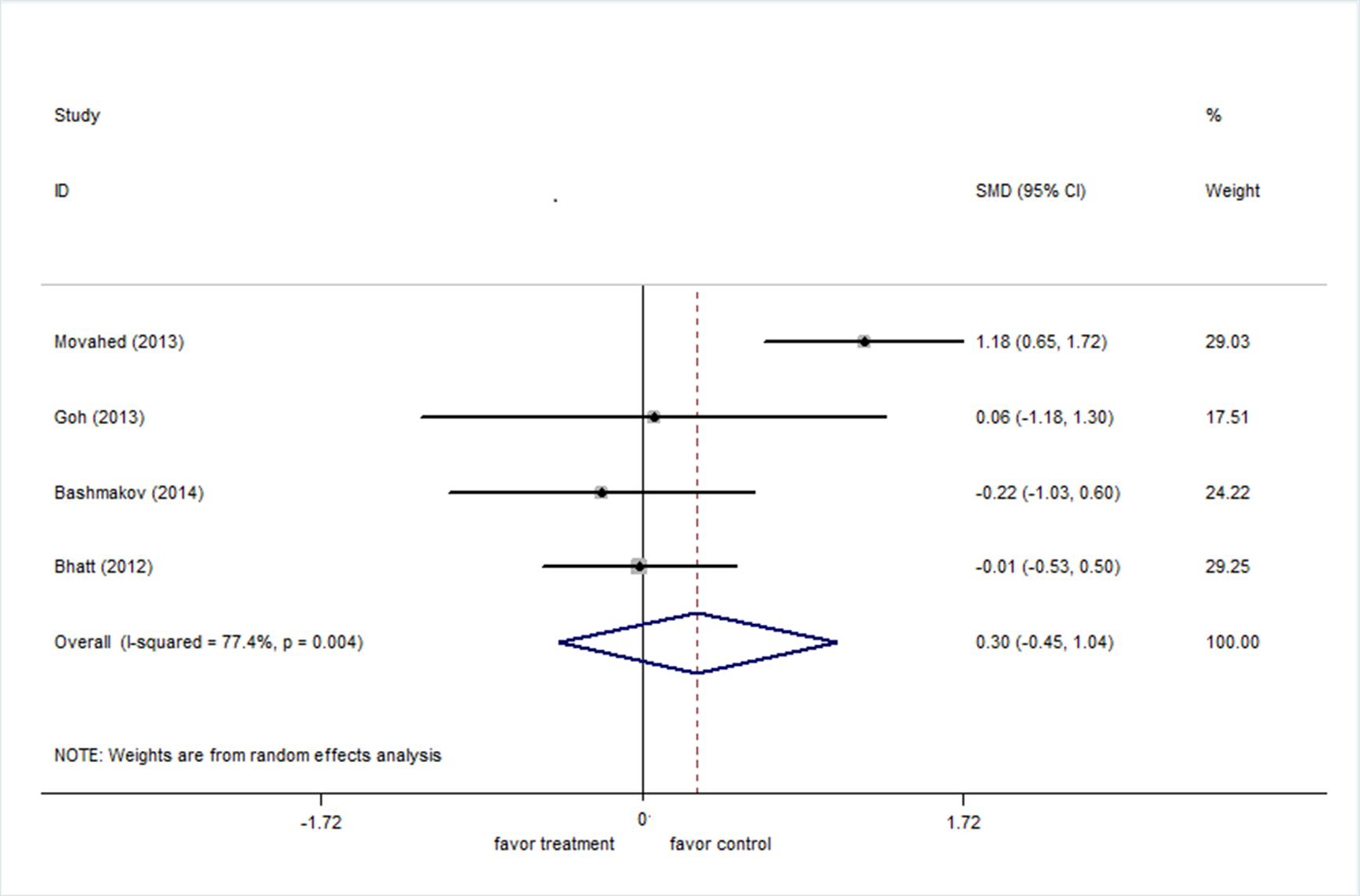

Supplement: Supplementary file 2 — Funnel plots of meta-analysis of the effect of resveratrol on other parameters. Forest plot of effect of resveratrol on insulin level. Forest plot of effect of resveratrol on systolic blood pressure. Forest plot of effect of resveratrol on diastolic blood pressure. Forest plot of effect of resveratrol on low-density lipoprotein cholesterol. Forest plot of effect of resveratrol on high-density lipoprotein cholesterol. (ZIP 1369 kb) [file 12986_2017_217_MOESM2_ESM.zip › Figure_S1E.tif]

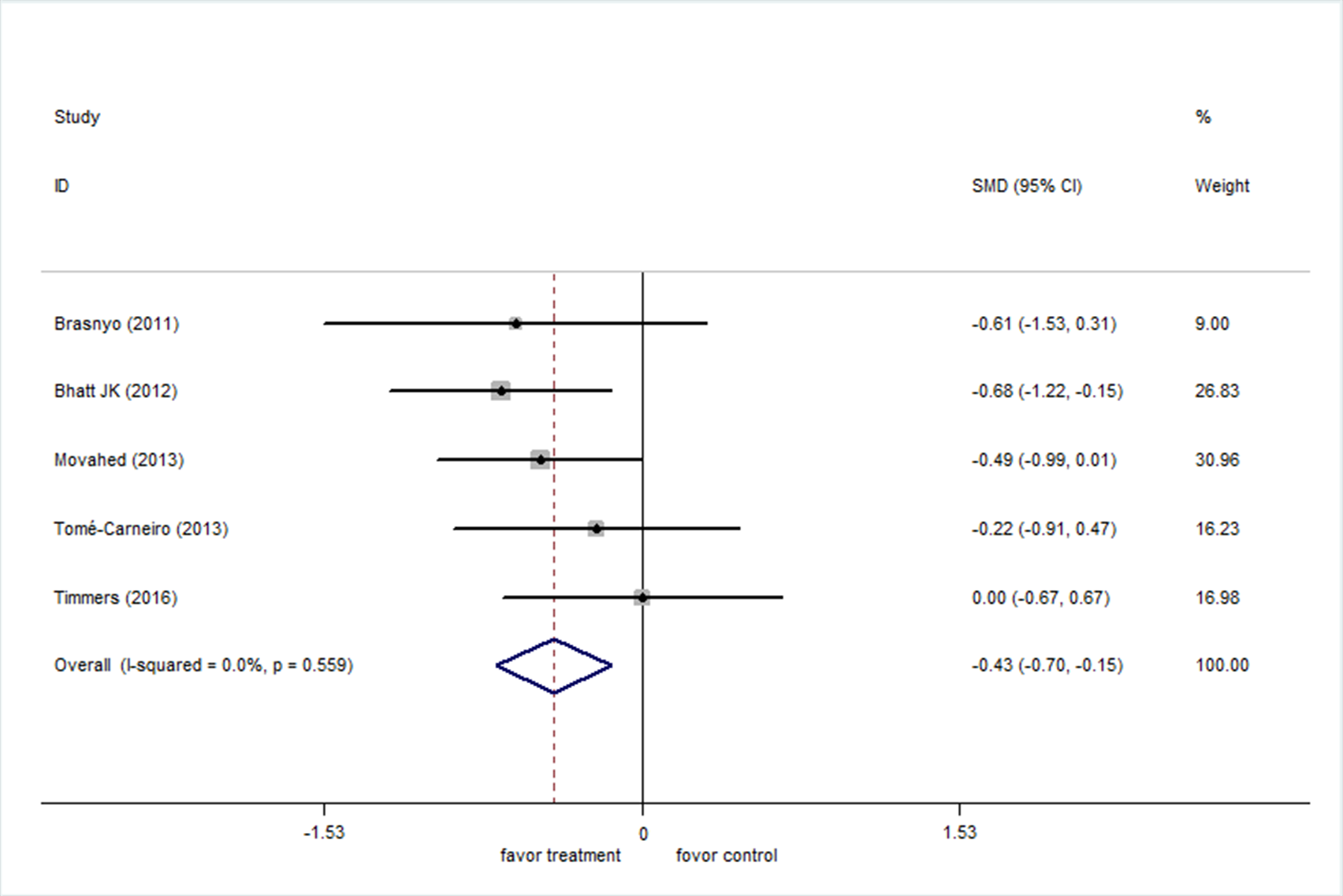

Supplement: Supplementary file 2 — Funnel plots of meta-analysis of the effect of resveratrol on other parameters. Forest plot of effect of resveratrol on insulin level. Forest plot of effect of resveratrol on systolic blood pressure. Forest plot of effect of resveratrol on diastolic blood pressure. Forest plot of effect of resveratrol on low-density lipoprotein cholesterol. Forest plot of effect of resveratrol on high-density lipoprotein cholesterol. (ZIP 1369 kb) [file 12986_2017_217_MOESM2_ESM.zip › Figure_S1C.tif]

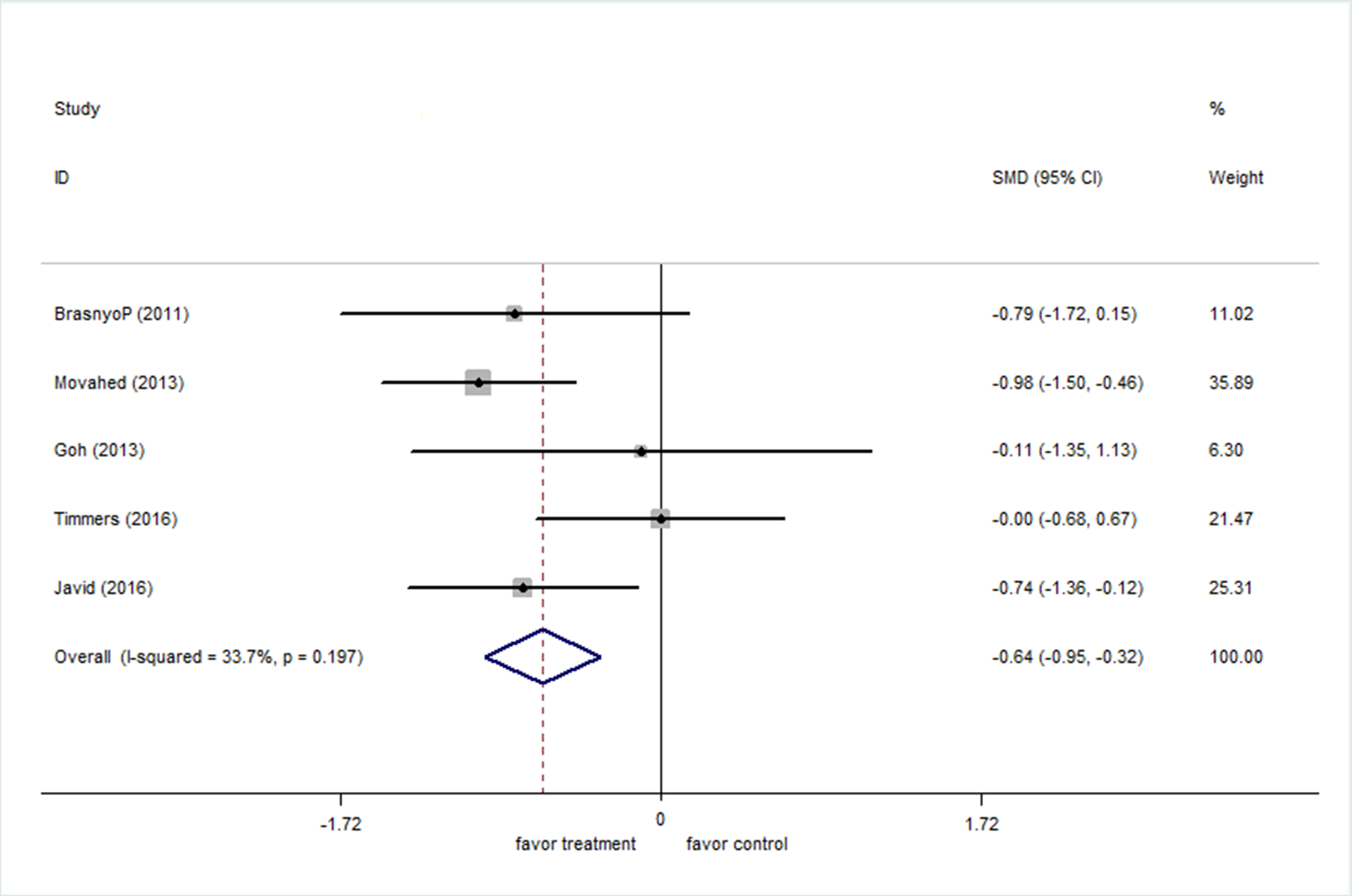

Supplement: Supplementary file 2 — Funnel plots of meta-analysis of the effect of resveratrol on other parameters. Forest plot of effect of resveratrol on insulin level. Forest plot of effect of resveratrol on systolic blood pressure. Forest plot of effect of resveratrol on diastolic blood pressure. Forest plot of effect of resveratrol on low-density lipoprotein cholesterol. Forest plot of effect of resveratrol on high-density lipoprotein cholesterol. (ZIP 1369 kb) [file 12986_2017_217_MOESM2_ESM.zip › Figure_S1A.tif]

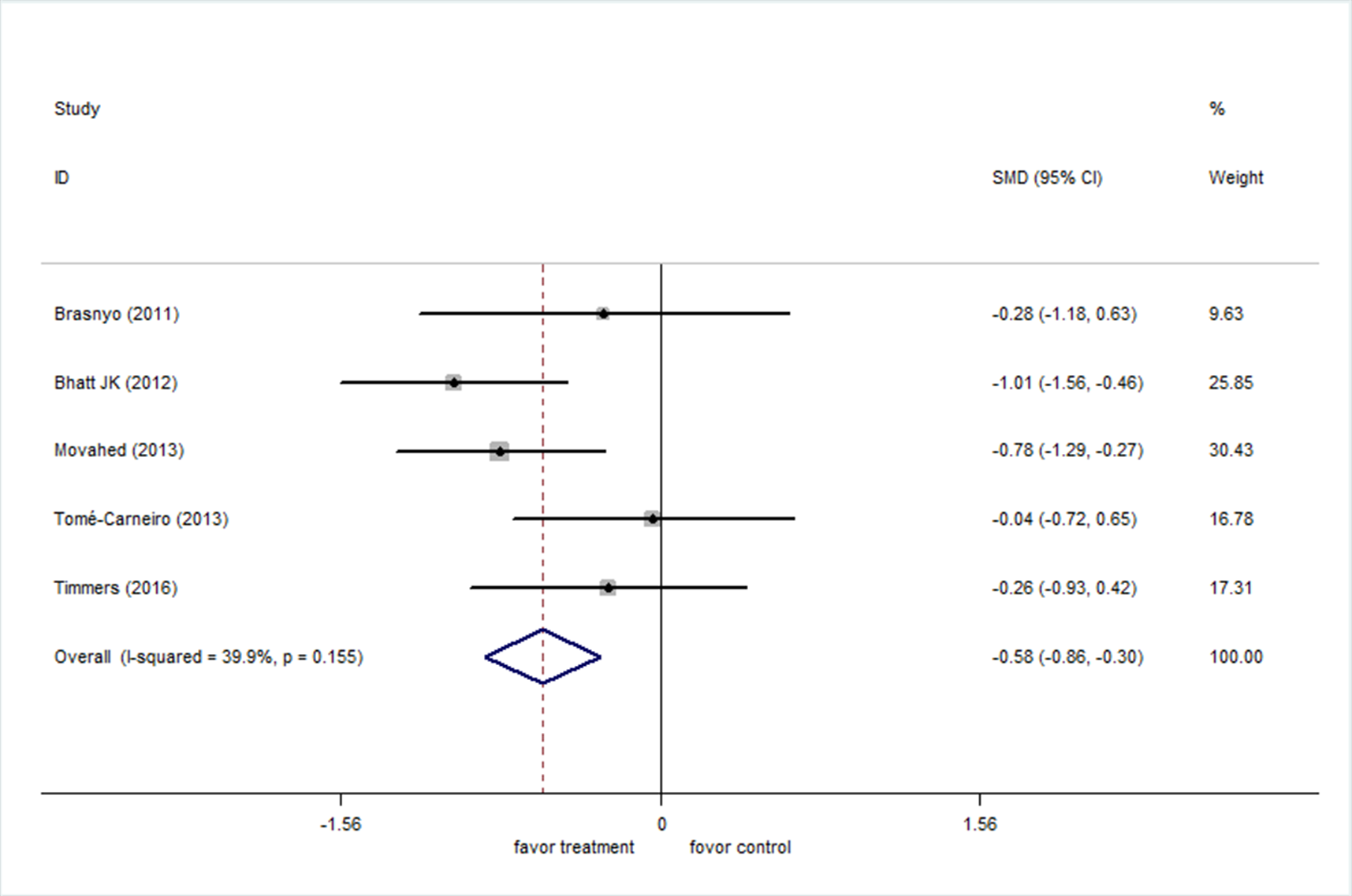

Supplement: Supplementary file 2 — Funnel plots of meta-analysis of the effect of resveratrol on other parameters. Forest plot of effect of resveratrol on insulin level. Forest plot of effect of resveratrol on systolic blood pressure. Forest plot of effect of resveratrol on diastolic blood pressure. Forest plot of effect of resveratrol on low-density lipoprotein cholesterol. Forest plot of effect of resveratrol on high-density lipoprotein cholesterol. (ZIP 1369 kb) [file 12986_2017_217_MOESM2_ESM.zip › Figure_S1B.tif]

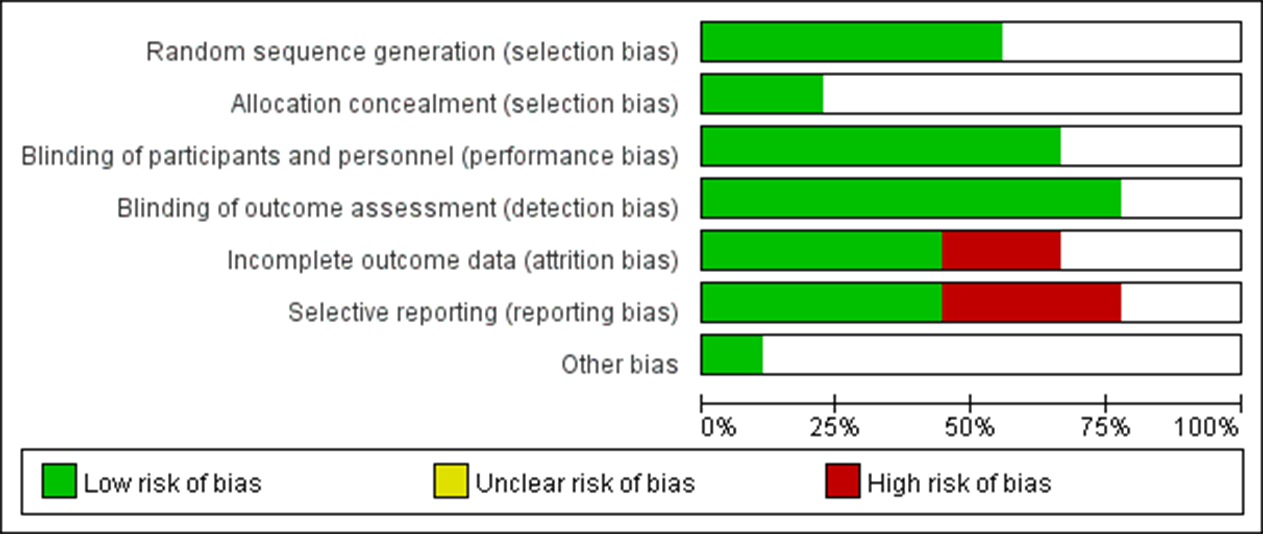

Supplement: Supplementary file 3 — Risk of bias of include trials. Random sequence generation: Unclear risk of bias in 4 trails for insufficient information about the sequence generation process. Allocation concealment: Unclear risk of bias for insufficient information in 7 trails. Blinding of participants and personnel: Unclear risk of bias for insufficient information in 3 trails. Blinding of outcome assessment: Unclear risk of bias for insufficient information in 2 trails. Incomplete outcome data: High risk of bias in 3 trails for unbalanced high proportion of dropped participants. Other bias: Low risk of bias in 1 trails. (TIFF 212 kb) [file 12986_2017_217_MOESM3_ESM.tif]
